# Adaptive working memory training does not produce transfer effects in cognition and neuroimaging

**DOI:** 10.1038/s41398-022-02272-7

**Published:** 2022-12-13

**Authors:** Isabelle Ripp, Mónica Emch, Qiong Wu, Aldana Lizarraga, Robert Udale, Claudia Christina von Bastian, Kathrin Koch, Igor Yakushev

**Affiliations:** 1 Department of Nuclear Medicine, School of Medicine, Klinikum Rechts der Isar, Technical University of Munich, Munich, Germany; 2 TUM-Neuroimaging Center (TUM-NIC), Technical University of Munich, Munich, Germany; 3 Department of Neuroradiology, School of Medicine, Klinikum Rechts der Isar, Technical University of Munich, Munich, Germany; 4 Institute of Medical Psychology, Ludwig-Maximilians-Universität, Munich, Germany; 5 Department of Psychology, University of Sheffield, Sheffield, UK

**Keywords:** Learning and memory, Psychology

## Abstract

Despite growing interest in cognitive interventions from academia and industry, it remains unclear if working memory (WM) training, one of the most popular cognitive interventions, produces transfer effects. Transfer effects are training-induced gains in performance in untrained cognitive tasks, while practice effects are improvements in trained task. The goal of this study was to evaluate potential transfer effects by comprehensive cognitive testing and neuroimaging. In this prospective, randomized-controlled, and single-blind study, we administered an 8-week *n*-back training to 55 healthy middle-aged (50–64 years) participants. State-of-the-art multimodal neuroimaging was used to examine potential anatomic and functional changes. Relative to control subjects, who performed non-adaptive WM training, no near or far transfer effects were detected in experimental subjects, who performed adaptive WM training. Equivalently, no training-related changes were observed in white matter integrity, amplitude of low frequency fluctuations, glucose metabolism, functional and metabolic connectivity. Exploratory within-group comparisons revealed some gains in transfer tasks, which, however, cannot be attributed to an increased WM capacity. In conclusion, WM training produces transfer effects neither at the cognitive level nor in terms of neural structure or function. These results speak against a common view that training-related gains reflect an increase in underlying WM capacity. Instead, the presently observed practice effects may be a result of optimized task processing strategies, which do not necessarily engage neural plasticity.

## Introduction

Working memory (WM) is the ability to retain temporary access to a limited amount of information in the service of ongoing cognitive processing [1]. The amount of accessible information is determined by WM capacity, which varies considerably between individuals [2]. WM capacity is closely correlated with other higher-order cognitive functions such as fluid intelligence, abstract reasoning, and reading comprehension [3]. This association motivated the development of cognitive training interventions targeting WM capacity in order to broadly improve general cognitive performance [4–7] and to counteract cognitive deficits in patient populations [8–10]. Moreover, WM training is a common component of cognitive training programmes that attract an increasing attention of major industry [11].

In the context of WM training, the concept of cognitive transfer is of key importance. Transfer effects are training-induced gains in performance in untrained cognitive tasks. Most previous theoretical accounts attributed transfer effects to training-induced increases in WM capacity, that is, a gain in the number of information elements that can be held accessible at the moment. Thus, transfer effects are an index of the putative effectiveness of WM training [12]. Transfer is categorized through the similarity between training and transfer tasks; the more similar the transfer task to the training task, the “nearer” the transfer. Hereafter, we refer to cognitive improvements in the trained task as practice effects, improvements in contextually highly similar WM tasks as nearest (sometimes also referred to as direct) and gains in dissimilar WM tasks as near transfer effects. Improvements in other yet related cognitive domains such as reasoning is referred to as far transfer. The main goal of WM training is to produce far transfer effects that could manifest in improved skills of daily functioning.

**Fig. 1:**
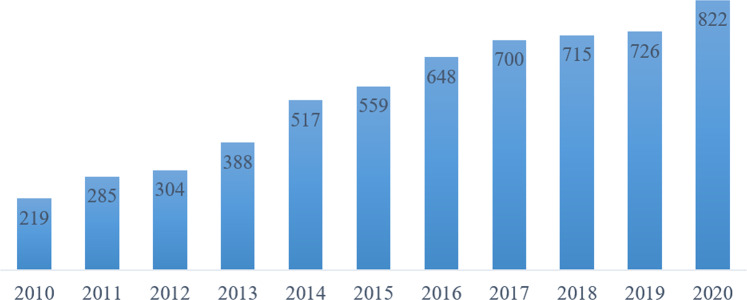
Number of studies published annually in the past decade. The studies were retrieved from PubMed using key words « “working memory”, training ».

The prospect of broadly improving cognitive performance through WM training has given rise to an increasing number of WM training studies published each year (Fig. 1). Meta-analyses averaging effects of training on WM tasks with varying similarity have reported significant near transfer effects, e.g., [13, 14]. However, more recent meta-analyses that distinguished between nearest and near transfer effects found significantly greater effect sizes for the former, suggesting that WM training may mainly yield task-specific transfer rather than a general improvement in WM [5, 15]. Regarding far transfer, meta-analyses have yielded inconsistent conclusions. For example, the meta-analysis of Au et al. [16] reported a small, but significant positive transfer effect on fluid intelligence in healthy young adults. However, subsequent meta-analyses did not find any significant far transfer effects [5, 13, 17], especially when the effects of WM training were compared with an active control group, that is, a group practicing tasks with little WM demands. The presence or absence of an active control group has been proposed as a key reason of the discrepant findings [6]. Furthermore, differences in the classification of cognitive tasks, training intensity, and type of training tasks have been discussed as potential reasons of the inconsistencies in previous findings (for a more detailed discussion, see von Bastian & Oberauer [7]). In addition to variations across training regimes, the validity of the assessment of cognitive outcomes has been criticized, too (e.g., [18]). Specifically, measurement noise and impurity of cognitive tasks may mimic or obscure true underlying changes in cognitive abilities.

Whereas a passive (or no-contact) control group accounts for test-retest effects, only an active control group accounts for non-specific training effects such as engaging regularly in a computerized task, expectancy, and placebo effects [7]. Moreover, practicing WM tasks can also trigger cognitive changes in domains different to WM capacity, such as attention and visual integration [19], or the development of task- or material-specific expertise [20]. Therefore, to resolve the controversies around the effectiveness of WM training, it is essential to discriminate between transfer driven by increased capacity in the trained domain such as WM capacity, and by enhanced use of the existing capacity [7]. This distinction is possible by including an active control group.

Neuroimaging complimentary to cognitive assessments has the potential to pick up training-induced neural plasticity, which can be assumed to constitute the basis of sustained cognitive improvement. Specifically, neuroimaging might depict evidence of neural plasticity that precedes measurable changes in cognition [21]. Furthermore, acquisition of both neuroimaging and cognitive markers potentialy offers a more comprehensive picture of the process under the study. Neural plasticity is a blanket term for acquired or learned changes in neural oscillations, myelin reformation, and synaptogenesis [22–24]. These processes can be indirectly measured, for example using functional magnetic resonance (fMRI), diffusion tensor imaging (DTI), and positron emission tomography with F18-fluordesoxyglucose (FDG-PET). The different forms of neural plasticity may occur simultaneously, and neuroimaging methods can provide only rough proxies of these changes at the macroscopic level [25]. Because it is still unknown which neuroimaging modality is most sensitive to training-induced neural plasticity, we followed an exploratory multi-modal approach in this study.

So far, most imaging studies of the neural substrates of cognitive gains induced by WM training have used task-related fMRI. However, many such studies focused on neural correlates of practice rather than transfer effects (for a review, see ref. [26]). Moreover, to the best of our knowledge, only one study has investigated effects of WM training on the magnitude of intrinsic neural activity at rest [27]. The authors found increased activity in the right dorsolateral prefrontal cortex, frontopolar area, and medial prefrontal cortex. Recently, the focus of neuroimaging studies has moved from voxel-wise analyses of signal amplitude to network-wise analyses, with resting-state networks (RSN) becoming particularly popular. Yet, only two fMRI studies have investigated resting-state functional connectivity changes in relation to WM training [28, 29]. Using a priori defined ROIs or networks of interest, these two studies reported connectivity changes, both increases and decreases, within and between regions of the frontal parietal and the default mode network (DMN). However, neither of these studies used an active control group nor did their analyses cover the whole brain. Similarly, only a few studies have investigated changes in structural connectivity, that is, neural tracts, following WM training [30–33]. Results indicated training-associated changes in frontal and parietal white matter tracts [30, 33] or the corpus callosum [31]. Takeuchi et al. [32] reported that WM training increased the mean diffusivity in regions of the dopaminergic system. Finally, although some FDG-PET studies investigated neural correlates of mental exercise [34–36], no such PET study exists in conjunction with WM training. This is surprising given that the concept of neurometabolic coupling underlying FDG-PET [37] is thought to be mediated by changes in neuro-glial energy pathways that support synaptogenesis or synaptic plasticity (for a review see ref. [38]).

The goal of this study was to investigate cognitive and neural effects of an 8-week adaptive *n*-back training intervention in healthy middle-aged participants. N-back training is one of the most extensively studied forms of WM training [5]. Importantly, a meta-analysis reported a trend for n-back being the most efficient WM training [13]. We focused on middle age due to the clinical relevance of this age group. Specifically, middle age directly precedes aging that is associated with a number of cognitive disorders such as Alzheimer’s disease. Yet, different to older adults, healthy middle-aged subjects typically have no significant atrophy or vascular pathology that might otherwise interfere with WM-related neural plasticity [39]. Therefore, should the current WM training programme prove effective, it might serve as an intervention in older adults to delay subsequent age- or disease-associated cognitive decline. To control for expectancy and non-specific cognitive intervention effects, we compared n-back training to an active control training with low cognitive demand. We used state-of-the-art hybrid PET/MRI equipment for simultaneous acquisition of MRI and PET data at baseline and after WM training. All neuroimaging data were analyzed at the whole-brain level using both voxel- and network-wise approaches.

Given the above literature, we expected to find at least near transfer effects and to detect changes at least in fMRI-RSNs as temporarily most dynamic/plastic and energetically least consuming imaging marker.

## Participants and methods

### Participants

The study was approved by the Federal Office for Radiation Protection and the local ethics review board (project number 399/13). Participants were recruited via advertisements in the internet and on hospital bulletin boards. Participants were right-handed, 50–64 years old, and free of cognitive deficits, neurological and psychiatric diseases. Further inclusion criteria were the absence of contraindications for MRI and no brain anomalies on structural MRI images. All participants provided written, informed consent. They were randomly assigned single-blinded to an experimental or an active control group. Among initially recruited participants 7 were excluded: two due image artefacts from large falx ossifications on MRI, one due to excessive head motion, one due to a failure to follow the instructions of the training programme; imaging data of two subjects were saved incompletely; one participant dropped out for personal reasons after the first neuroimaging session. Finally, data of 55 participants (30 males) with a mean age of 55.9 years (SD = 4.2 years) were available for further analyses: 28 in the experimental and 27 in the active control group.

### Working memory training

**Fig. 2:**
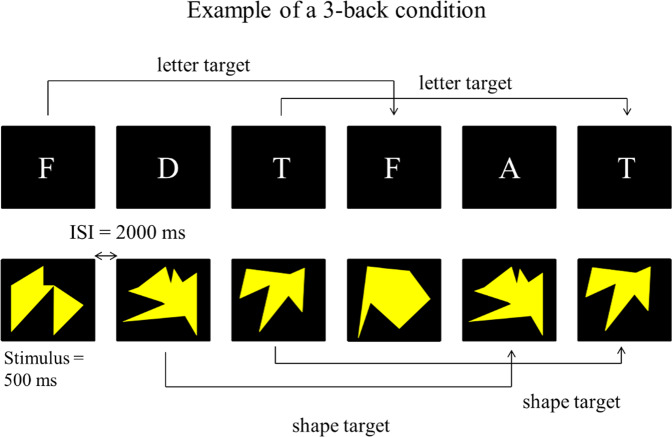
Example of a verbal 3-back level (top) and a visual 3-back level (bottom). ISI interstimulus interval.

All participants underwent supervised training on a personal computer at home. Training in both the experimental and the active control group consisted of variants of visual and verbal *n*-back tasks. In these tasks, participants were presented a sequence of stimuli and were asked to identify a target stimulus. Targets were stimuli matching the stimulus shown *n* positions back (Fig. 2). Each stimulus was presented for 500 ms followed by a 2000 ms interstimulus interval. Participants were instructed to press the “A” key in response to target stimuli.

The experimental group performed adaptive visual and verbal *n*-back tasks adapted from Jaeggi et al. [40]. In each training session, participants completed 9 blocks per task (18 training blocks in total). Each block included a randomized sequence of 6 targets and 14 non-target stimuli. Task difficulty (i.e., the *n* positions that targets had to be matched against) was adapted to the individual performance based on the proportion of correct responses computed as a sum score of hits (i.e., correctly detected targets) and correct rejections of non-targets. The *n*-back level of the subsequent block increased if the proportion of correct responses was greater than 90% and decreased if it was lower than 80%. Otherwise the *n*-back level remained the same. The adaptive level of *n* ranged from 1 to a maximum difficulty level of 9. Only one participant reached the 9-back level, indicating the absence of a ceiling effect at the group level.

The active control group performed a non-adaptive low-level training intervention with visual (X-back) and verbal (1-back) tasks with task structures and stimuli equivalent to the adaptive *n*-back training tasks. In the X-back task, participants had to press the “A” key whenever a target shape was presented. Hence, the X-back task demanded primarily attentional processing but no working memory. The same target shape was shown at the beginning of each block in all training sessions. The verbal 1-back task was identical to the adaptive verbal *n*-back task except that the level of *n* remained at 1 regardless of individual performance. Like the experimental group, the active control group completed 9 blocks of each task in each session, with each block consisting of sequences of 6 target and 14 non-target stimuli for the visual X-back task, and 6 targets and 14 non-target stimuli in the verbal 1-back task.

The order of tasks trained (visual or verbal) was counterbalanced between participants within each group. Each training session took approximately 20 min. Participants were instructed to complete four training sessions per week and one training session per day. After each training session, logfiles were automatically uploaded to the Millisecond Software website (https://www.millisecond.com/). Based on information saved in the logfiles, a weekly training progress report was sent via email to all participants. In case of irregularities in training behaviour, e.g., incompleteness of training sessions, we reminded the participant to follow the training instructions carefully.

### Cognitive test battery

A test battery was administered one week before the start and one week after the end of the WM training intervention. Before the first cognitive assessment, each task was explained by an experimenter, and the participants completed a few practice trails in the presence of an experimenter. Each cognitive assessment lasted approximately 80 min. Three tests were used to assess nearest transfer effects to untrained WM measures: Digit Span test for verbal WM (forward and backward version; subtest from HAWIE-R); Simple Visual Reaction Time (SVRT) task for motor response velocity and attention [41], and Corsi-Block Tapping test for visual WM [42]. Performance assessment was based on scores for each subtest of the Digit Span test, on a mean latency value from the SVRT task, and a block span of the Corsi-Block Tapping test [43].

Near transfer effects to tasks measuring attention and learning were assessed using visual Simon task (VST, [44]) and Colour Word Stroop task (CWST, [45]). For both near transfer tasks, we recorded correct responses for incongruent and congruent trials separately. Simon task trials were categorized as congruent if location of the stimulus was equivalent to location of the response key on the laptop and incongruent if locations of the stimulus and the response key were opposite. Colour Word Stroop task trials were categorized as congruent if meaning of the word and its colour aligned and incongruent otherwise. In addition, we administered a short-term memory test (Verbaler Lern- und Merkfähigkeitstest, VLMT, [46]) and a sustained attention test (Rapid Visual Information Processing, RVIP, [47]). For the VLMT, we analyzed three dependent variables: difference in the number of correct answers between the recall before and after presentation of the interference list (Dg5-Dg6), difference in the number of correct answers between the recall before and 20–30 min after presentation of the interference list (Dg5-Dg7), and scores from the Word Recognition List (WR). For the RVIP, we analyzed the proportion of correct target detections.

To assess far transfer effects to fluid intelligence and decision-making, we used a short version of the Raven’s Advanced Progressive Matrices Test (short-APM, [48], and the Iowa Gambling Task [49], respectively. Performance in the short-APM was scored by the number of correct responses, whereas performance in the Iowa Gambling Task was ranked by the net score (good play–bad play).

The Digit Span task and VLMT were presented orally, whereas the other tests were administered on a PC with an in-house adaptation of the Millisecond website test library (Inquisit 5; retrieved from: https://www.millisecond.com). The short-APM was coded in the Inquisit programming language. The Digit Span forward requires the participant to repeat digits in the same order as presented by the examiner. A minimum length of three and a maximum length of nine digits are presented. The Digit Span backward requires the participant to repeat the digits in the reverse order as presented by the examiner. Here, a minimum of two and a maximum of eight digits is presented. The number of digits increases when the participant correctly repeats at least one out of two trials.

### Imaging data acquisition

Imaging data were acquired on a 3T hybrid PET/MR Siemens Biograph mMR scanner with a vendor-supplied 16-channel head coil. The subjects were instructed to fast for six hours prior to each of two PET/MR sessions. Around 100 MBq FDG were injected intravenously to participants sitting in a quiet, dimly lit room, after confirming normal blood glucose levels. The following MR sequences were acquired over the first 30 min of imaging (i.e., 30–60 min post injection, p.i.): localizer, μ-map, structural T1-weighted, FLAIR, echo-planar imaging (EPI) 2D diffusion for diffusion tensor imaging (DTI) and EPI- Prospective Acquisition Correction sequence for resting state functional MRI (rsfMRI). For rsfMRI participants were instructed to close their eyes and think of nothing in particular. Task fMRI underlying visual and verbal n-back task was acquired 60–90 min p.i. These data were published previously [50]. For each subject, we reconstructed a single frame FDG-PET summation image for 30–60 min p.i. Detailed parameters of PET acquisition and MR sequences are described in Supplementary Material. The same imaging protocol was used in both sessions. The presence of significant microangiopathic lesions and incidental findings were excluded upon visual assessment of structural MRI images.

### Imaging data analysis

DICOM files were converted to 3D-NIFTI volumes using the dcm2niix tool (https://github.com/neurolabusc/dcm2niix), except for the fMRI data, for which we used dm2nii. FMRI and FDG-PET data were pre-processed using SPM12 (https://www.fil.ion.ucl.ac.uk/spm/software/spm12/) and MATLAB v2017b (The MathWorks Inc., Natick, Massachusetts, USA). DTI data were pre-processed using the University of Oxford’s Centre for Functional Magnetic Resonance Imaging of the Brain Software Library (FSL) version 6 (http://www.fmrib.ox.ac.uk/fsl/index.html). PET images were spatially normalized into the MNI space using a study-specific FDG-PET template, followed by smoothing with an 8 mm isotropic Gaussian filter. The first three images of the fMRI data were discarded. Data preprocessing included motion correction, coregistration of the subjects’ T1-weighted image to the functional images, spatial normalization to the MNI space using DARTEL, and smoothing with an 8 mm isotropic Gaussian filter. Excessive head motion was defined as translation >3 mm or rotation >3° [51].

The amplitude of low frequency fluctuations (ALFF) analysis was carried out using Data Processing Assistant for Resting-state fMRI (http://rfmri.org/dpabi) and SPM12. The pre-processed and smoothed rsfMRI data (see above) were further processed using linear de-trending, nuisance regression (i.e., white matter signal, cerebrospinal fluid signal, 6 motion parameters and their first derivatives) and band-pass filtering (0.01–0.08 Hz) to remove low-frequency drifts and other high-frequency physiological noises. Then, ALFF maps were calculated as described previously [52]. In brief, the filtered time series were transformed into the frequency domain with fast-Fourier transform. Then, the square root of the power spectrum was computed and averaged at each voxel.

Following a visual inspection, passed DTI images were corrected for susceptibility-induced distortions, eddy currents, subject movement, and signal dropout using the tool Eddy [53]. Brain tissue was derived using the brain extraction tool (https://fsl.fmrib.ox.ac.uk/fsl/fslwiki/BET). Images of four subjects, three experimental and one control, had to be excluded from further DTI analyses due to an incorrect phase encoding direction. To obtain eigenvalues L1 (axial diffusivity, AD), L2, and L3 with corresponding eigenvectors, as well as maps of fractional anisotropy (FA) and mean diffusivity (MD), a diffusion tensor model was fitted at each voxel using FSL’s DTIFIT https://fsl.fmrib.ox.ac.uk/fsl/fslwiki/FDT. Additionally, radial diffusivity (RD) maps were created by averaging the L2 and L3 maps. Individual FA maps were spatially normalized to the MNI space. A mean FA map was used to compute an average white matter tract skeleton using a threshold of FA > 0.2. Finally, Tract-Based Spatial Statistics as implemented in FSL was conducted for FA, MD, AD, and RD maps.

### Independent component analysis

We applied a spatial independent component analysis (ICA) to the rsfMRI data and a spatially constrained ICA to the FDG-PET data using GIFT toolbox v3.0b (Medical Imaging Analysis Lab, The Mind Research Network; http://mialab.mrn.org/software/gift). Individual fMRI time-series images were concatenated for the group ICA using the Infomax algorithm [54]. We chose a 30 component ICA model, as this intermediate model order delivers robust and coherent RSNs [55]. We applied the resulting spatial maps as reference templates for the spatially constrained ICA applied to the FDG-PET data. Hereby, a concatenation of one PET image per participant was used for the group ICA [56–58], while employing the same brain mask as for fMRI ICA. Further details on the spatially constrained ICA for the FDG-PET data can be found in the Supplementary Materials. We focused our analyses on the following (neurocognitive) networks of interest a priori: anterior and posterior DMN, salience network (SN), and left and right central executive network (CEN). The auditory network was chosen as reference network, as it was assumed to be unaffected by visual and verbal *n*-back training.

### Indices of network integrity

Indices were calculated both for fMRI- and FDG-PET-based RSNs. For the fMRI data, we calculated a multiple regression against the group-derived component maps using the function “icatb_multipleRegression” of the GIFT toolbox. This analysis returned a beta-coefficient (β) value for each component of interest in each participant, reflecting the degree to which the spatial pattern of each participant’s particular network explained the spatial pattern of the equivalent group-derived network. For this computational step, we used a z-threshold of 1 for the reconstructed participant-specific component maps [59]. For the FDG-PET data, we extracted so called loading coefficients [58, 59]. These are the mixing-matrix entries of A from the generative model x = As that separate different signals [60, 61]. These values were read out from the estimated “timecourse” file, with each timepoint representing one participant (see above). Loading coefficients close to zero represent a high spatial overlap between each participant’s RSN and the equivalent group-derived RSN. Finally, indices of network integrity were available for each participant, network, and imaging modality [59]. Potential WM training effects on network integrity were analyzed with a two-way mixed-effects analysis of variance (ANOVA) for repeated measures using the between-subjects factor *group* (CON, EXP) and the within-subjects factor *time* (T1, T2) in SPSS 19.0 (IBM Corporation, Somers, NY). We considered results as statistically significant at *p* < 0.05 after Bonferroni correction, i.e., 0.05/6 = 0.008 for six networks of interest.

### Statistical analyses of the training data

We used in-house written Python 3 scripts to analyze the training data. In the experimental group, we studied the mean *n*-back level achieved in each session (experimental group only) and the *d* prime (both training groups). Based on signal detection theory, *d* prime is calculated as the difference between the hit rate and the false alarm rate [62]. For the control group, we analyzed only *d* prime, since those participants performed at the same low *n*-back level throughout training. Because the last three sessions of one participant in the experimental group and two last sessions of one participant in the control group were lost, we interpolated the missing data with their own previous training data using a forward linear method. Assumptions of normality were rejected for the *n*-back training data (all *p*-values < 0.05 in Kolmogorov–Smirnov test). Therefore, to assess practice effects, we performed a two-sided Wilcoxon signed-rank test between the mean of the first four and the mean of the last four training sessions for *d* prime, separately for each group and WM training modality (i.e., visual and verbal). In addition, we computed a two-sided Wilcoxon signed-rank test between the mean of the first four and the mean of the last four sessions for *n*-back level values for the experimental group separately for each training modality (i.e., visual and verbal).

### Statistical analyses of the cognitive test battery

Assumptions of normality were tested using a one-sample Kolmogorov–Smirnov test. As primary analysis, we conducted a *group* (CON, EXP) by *time* (T1, T2) multivariate ANOVA for each transfer category (i.e., nearest, near, far). In case of a significant *group x time* interaction per category, we performed a post hoc ANOVA for this category. Results were considered as statistically significant at *p* < 0.05 with Bonferroni correction, that is, 0.05/4 = 0.0125; 0.05/15 = 0.003; 0.05/2 = 0.025 for 4, 15, and 2 tests for nearest, near and far transfer, respectively. ANOVA is known to be robust to not normally distributed data with an equal sample size [63]. In addition, we computed two-sided paired *t*-tests or Wilcoxon signed-rank tests (depending on the distribution) for all cognitive tests within the experimental and control group (Table 3). Results were considered as statistically significant at *p* < 0.05 with Bonferroni correction, that is, 0.05/21 = *p* < 0.002 (for 21 tests). All statistical analyses were performed using SPSS 19.0 (IBM Corporation, Somers, NY). Moreover, we analyzed cognitive data assessing transfer effects with Bayesian statistics. The Bayesian analysis quantifies confidence in a model in the face of the data. The Bayes factor is the comparison of posterior likelihoods of competing models. Bayes factors are the relative posterior likelihood of the data under one model (such as the null) against a competing model (such as the alternative). The advantage of this approach is that, unlike null-hypothesis significance testing, Bayes factors are informative about whether there is sufficient evidence to accept or reject one model over the other, or whether further data collection is needed [64]. The Bayes factor is a ratio with its magnitude (ranging from 0 to ∞) providing a continuous measure of the strength of evidence in favour of the numerator model over the denominator model. Taking the reciprocal of a Bayes factor gives the strength of evidence in favour of the denominator model. A Bayes factor of 1 reflects perfectly ambiguous evidence (i.e., the data is not sufficiently sensitive to distinguish between the two hypotheses), with larger Bayes factors representing stronger evidence. Conventionally, Bayes factors below 3 are deemed as reflecting ambiguous evidence. We replicated the frequentist analysis with Bayesian t-tests and ANOVAs where appropriate. The null and alternative models were specified with prior probability distributions, which quantify one’s beliefs under the model over different effect sizes before observing the data. We used a set of widely accepted default priors for *t*-tests [65] and ANOVAs [66]. For ANOVAs these were: fixed effects: *r* = 0.5, random effects: *r* = 1, scale covariates: *r* = 0.354. For t-tests, the priors were: Cauchy prior with scale = 0.707. The analysis was run using JASP (JASP team, 2021). We reported the model averaged Bayes factors across matched models, with subscripts indicating which model was the numerator (1 = alternative, 0 = null). Evidence for or against transfer was assessed using the model-averaged Bayes factor for the two-way interaction between group and time.

### Statistical analyses of the imaging data

For the ALFF and FDG-PET data we conducted ANOVA with the between-subjects factor *group* (CON, EXP) and the within-subjects factor *time* (T1, T2) using the full factorial model in SPM12. For the FDG-PET data grand-mean scaling and global calculation using SPM’s global mean were applied. A *p* < 0.05 familywise error corrected at a voxel-level was set as the significance threshold. To analyze the DTI data voxel-wise, we applied permutation-based statistics using the *randomize* function in FSL [67]. The random permutation number was set at 5000, and we considered results as statistically significant at *p* < 0.05 threshold-free cluster enhancement, again corrected for multiple comparisons at a voxel-level. Two-way mixed-effect ANOVA for repeated measures was conducted with the between-subjects factor *group* (CON, EXP) and the within-subjects factor *time* (T1, T2) for FA, MD, AD, and RD maps. For explorative reasons, we also present all results with an uncorrected *p <* 0.001 with a cluster extent threshold of 50 for the voxel wise analyses of the ALFF, FDG-PET data, and TBSS.

## Results

### Demographics

Demographic characteristics of the participants are summarized in Table 1. There was no significant difference for age (*p* = 0.92), sex (*p* = 0.70), BMI (*p* = 0.19), or years of education (*p* = 0.38) between the experimental and the active control groups. Thus, no correction for these variables was applied [68].

**Table 1:** Demographics.

	N	M/F	Age	BMI	YoE
Experimental	28	16/12	56.00 ± 4.23	25.83 ± 4.15	16.79 ± 3.14
Active control	27	14/13	55.88 ± 4.23	24.49 ± 3.33	16.01 ± 3.21

*M* male, *F* female, *BMI* body mass index, *YoE* years of education; data presented as mean ± standard deviation; two-sided two sample *t*-tests was applied for age, body-mass index, and years of education; chi-squared test was applied for sex.

### Working memory training

**Fig. 3:**
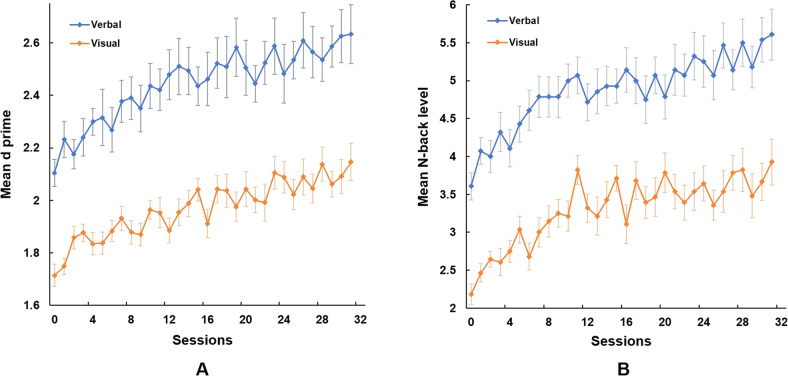
Training results for the experimental group. **A** *d* prime mean values per session, **B** *n*-back level mean values per session; data are shown as mean ± SEM.

The experimental group showed significant practice effects both in verbal *n*-back training (*Z* = 7, *p* = 8.07e−6) and visual *n*-back training (*Z* = 13, *p* = 1.51e−05), Fig. 3A. It also showed a significant improvement in the *n*-back level achieved in verbal *n*-back training (*Z* = 21, *p* = 3.34e−05) and in visual *n*-back training (*Z* = 18.5, *p* = 6.51e−05), Fig. 3B. In the active control group, *d* prime did not significantly differ between the beginning and the end of verbal *n*-back training or visual *n*-back training.

In the multivariate ANOVA, a significant *group* × *time* interaction was found only for the nearest transfer effect category (F_(4,50)_ = 5.3, *p* < 0.013). Follow-up univariate analysis of variance revealed a significant *group × time* interaction for the Digit Span forward test, Table 2. The experimental group showed a significant improvement in the Digit Span forward test at T2 compared to T1, Table 3. Subjects in both groups showed quicker reaction times in the Visual Simon task (VST) at T2 compared to T1. Here, the experimental group improved in the incongruent trials (RT-incong), whereas the control group improved both in congruent and incongruent trials.

**Table 2:** Univariate ANOVA for all transfer categories.

WMT effects	Tests	WMT group		Active control group		Interaction group × time		Bayes factors statistics
		Pre M (SD)	Post M (SD)	Pre M(SD)	Post M (SD)	*F*	*P* (η2)	BF
Nearest transfer (3)	Digit Span							
	Forward	7.79(2.15)	8.82(1.7)	7.52(2.2)	7.00(1.8)	**16.97**	<0.0001 (0.243)*	**182.42**^a^
	Backward	6.75(1.3)	7.46(2.1)	7.11(2.4)	7.63(2.5)	0.11	0.75 (0.002)	3.46^b^
	Corsi	46.1(16.5)	47.68(16.5)	44.1(19.1)	44.7(16.4)	0.04	0.85 (0.001)	3.77^b^
	SVRT							
	M-latency	286.71(32.1)	306.32.(48.6)	292.64(50)	287.58(42.2)	6.16	0.02 (0.104)	3.31^**a**^
Near-transfer (4)	VLMT							
	Dg5–6	1.68(1.7)	2.11(2.9)	1.04(1.8)	1.00(1.4)	0.38	0.54 (0.007)	3.13^**a**^
	Dg5–7	1.25(1.6)	1.14(1.7)	0.70(1.5)	0.96(1.2)	0.64	0.43 (0.012)	2.75^**a**^
	w-f	12.64(2.5)	12.93(2.3)	13.44(1.5)	13.89(1.5)	0.09	0.76 (0.002)	3.66^**a**^
	RVIP							
	Accuracy	18.04(7.3)	20.79(5.0)	16.52(9.0)	19.89(5.7)	0.11	0.74 (0.002)	3.71^b^
	RT	523.97(76.12)	531.44(66.7)	508.63(83.2)	493.3(66.5)	1.39	0.24 (0.026)	1.98^b^
	CWST							
	RT-cong	1344.4(272.5)	1253.2(2643.)	1336.4(271.9)	1312.7(305.9)	1.21	0.28 (0.022)	2.16^b^
	RT-incong	1600.1(286.7)	1506.1(320.2)	1531.1(292.9)	1537.4(304.9)	1.67	0.20 (0.031)	1.83^b^
	RT-neutral	1266.5(221.7)	1191.3(233.3)	1228.8(237.2)	1234.9(265.5)	2.04	0.16 (0.037)	1.62^b^
	%-cong	0.996(0.01)	0.988(0.03)	0.994(0.02)	0.996(0.01)	1.86	0.18 (0.034)	1.55^b^
	%-incong	0.939(0.07)	0.931(0.07)	0.950(0.06)	0.948(0.05)	0.09	0.76 (0.002)	3.99^b^
	%-neutral	0.983(0.04)	0.990(0.03)	0.998(0.01)	0.995(0.01)	1.14	0.29 (0.021)	2.39^b^
	VST							
	RT-cong	454.63(59.1)	426.72(57.2)	447.49(63.0)	406.56(80.0)	0.99	0.32 (0.018)	2.42^b^
	RT-incong	505.13(64.9)	471.1(59.6)	492.74(71.8)	451.43(76.0)	0.56	0.46 (0.010)	2.97^b^
	%-cong	0.98(0.04)	0.99(0.02)	0.96(0.1)	0.98(0.02)	0.67	0.42 (0.012)	2.7^b^
	%-incong	0.963(0.04)	0.966(.03)	0.953(0.1)	0.943(0.06)	0.31	0.58 (0.006)	3.63^b^
Far-transfer (2)	Short- APM	5.64(2.30)	6.79(2.44)	5.26(2.49)	5.67(2.47)	1.99	0.16 (0.036)	1.78^b^
	IGT	6.21(8.32)	11.43(10.88)	5.46(3.19)	9.91(16.74)	0.03	0.87 (0.000)	3.99^b^

Notes: *WMT* working memory training, *M* (SD), mean (standard deviation), *Corsi* Corsi-block Tapping test; *SVRT* simple visual reaction time task, *M-latency* mean latency, *RVIP* the rapid visual information processing task, Accuracy = hits − FA; RT (ms), mean reaction time for correct responses in millisecond, *CWST* the colour-word stroop task, *RT-cong* mean reaction time for congruent condition, *RT-incong* mean reaction time for incongruent condition, *RT-neutral* mean reaction for neutral condition, *%-cong* percent correct for congruent condition, %-incong, percent correct for incongruent condition, *%-neutral* percent correct for neutral condition, *VST* visual Simon task, Short-APM, the short version of Raven’s Advanced Progressive Matrices Test; *IGT* Iowa Gambling Task, independent variable here is net score. *significant at *p* < .05 Bonferroni corrected, *BF* bayes factors; ^a^bayes factor in favour of H1 over H0; ^b^bayes factor in favour of H0 over H1.

The bold values indicate a statistically significant difference.

**Table 3:** Comparisons within each group for all cognitive tests.

	Tests	EXP (T1 vs. T2)			CON (T1 vs. T2)		
		*T*	*p*	BF_10_	*T*	*p*	BF_10_
Nearest transfer	Digit Span						
	Forward	*T* = −3.48	0.002**	20.97	*T* = 2.27	0.03*	1.78
	Backward	*T* = −1.54	0.14	0.57	*T* = −1.37	0.18	0.47
	Corsi-Block Tapping	*Z* = −0.16	0.25	0.22	*Z* = −0.30	0.76	0.21
	SVRT						
	Mean latency	*T* = −2.39	0.02*	3.45	*T* = 0.40	0.69	0.27
Near transfer	VLMT						
	Dg5–6	*Z* = −0.16	0.13	0.25	*Z* = −0.21	0.83	0.21
	Dg5–7	*T* = 0.31	0.76	0.21	*T* = −0.85	0.40	0.28
	w-f	*T* = −0.72	0.48	0.26	*T* = −1.35	0.19	0.46
	RVIP						
	Accuracy	*T* = −2.93	0.007*	6.34	*T* = −2.03	0.05	1.20
	RT	*T* = −0.54	0.59	0.23	*T* = 1.14	0.26	0.37
	CWST						
	RT-cong	*T* = 1.80	0.08	0.83	*T* = 0.70	0.49	0.25
	RT-incong	*T* = 1.43	0.17	0.50	*T* = −0.16	0.87	0.21
	RT-neutral	*T* = 1.67	0.11	0.68	*T* = −0.18	0.86	0.21
	%-cong	*Z* = −1.52	0.13	0.48	*Z* = −0.67	0.50	0.22
	%-incong	*Z* = −0.26	0.80	0.22	*Z* = −0.061	0.95	0.21
	%-neutral	*Z* = −0.76	0.45	0.27	*Z* = −0.56	0.56	0.32
	VST						
	RT-cong	*T* = 2.66	0.01*	3.99	*T* = 4.47	0.0001**	202.02
	RT-incong	*T* = 3.47	0.002**	11.93	*T* = 4.68	0.0001**	328.53
	%-cong	*Z* = −1.13	0.26	0.45	*Z* = −1.24	0.24	0.40
	%-incong	*Z* = −0.07	0.94	0.21	*Z* = −1.77	0.08	0.23
Far transfer	Short-APM	*T* = −2.95	0.006*	6.66	*T* = −1.17	0.25	0.38
	IGT	*T* = −2.00	0.056	1.12	*T* = −1.15	0.26	0.37

*Corsi* Corsi-Block Tapping test, *SVRT* simple visual reaction time task, *RVIP* the rapid visual information processing task, Accuracy *=* number of correct hits − FA, *RT* (ms) mean reaction time of correct target, in millisecond, *CWST* the colour-word Stroop task, *RT*-*cong* mean reaction time for congruent condition, *RT-incong* mean reaction time for incongruent condition, *RT-neutral* mean reaction in neutral condition, *%-cong* proportion correct for congruent condition, *%-incong* proportion correct for incongruent condition, *%-neutral*,proportion correct for neutral condition, *VST* visual Simon task, *Short-APM* the short version of Raven’s Advanced Progressive Matrices Test, *IGT* Iowa Gambling Task, independent variable here is net score. For all measures except RT and VLMT (Dg5-Dg6, Dg5-Dg7) negative T-values represent improvements in performance from T1 to T2. *T*-values for two-sided paired t-tests, *Z*-value for two sided Wilcoxon signed-rank test *Significant at *p* *<* 0.05 uncorrected; **significant at *p* *<* 0.05 Bonferroni corrected (*p* *<* 0.002 uncorrected); *BF*_10_, bayes factors.

Based on the Bayesian statistics, we found decisive evidence for nearest transfer effects to the forward digit span (BF_10_ = 182.42). Strong evidence from simple-effects analysis suggests that this was driven by a change in the experimental group (BF_10_ = 18.53) rather than in the control group (BF_10_ = 1.87). There was relatively weak evidence against transfer to the Corsi task (BF_01_ = 3.77). For near transfer, there was relatively weak evidence against transfer to the VLMT (BFs01: 2.75–3.66). For the RVIP, there was weak evidence against transfer in accuracy (BF_01_ = 3.71), and ambiguous evidence for transfer in RTs (BF_01_ = 1.98). For all measures of the VST and Stroop task, there was at least ambiguous evidence in support of the null (all BFs_01_ > 1.54). Finally, there was ambiguous evidence against transfer to the short-APM (BF_01_ = 1.78) and weak evidence against transfer in the Iowa Gambling Task (BF_01_ = 3.99).

### Voxel-wise analyses of the ALFF and PET data

The *group* × *time* interaction was not significant in both analyses. Explorative analyses with uncorrected thresholds (*p* < 0.001, *k* > 50 voxels) are shown in Supplemental Fig. S1. In brief, there were no plausible findings.

### Network analyses of the resting state fMRI and PET data

**Fig. 4:**
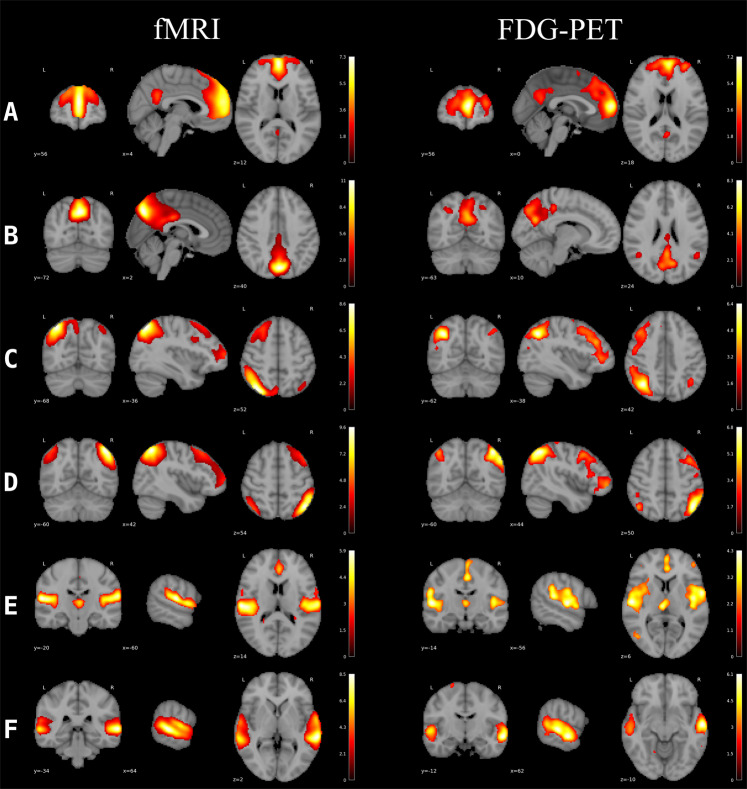
Resting state networks of interest in the PET data. Overlay of independent component maps onto the T1 template in the Montreal Neurologic Institute space at a threshold of *z* > 2.0. Colour bar represents *z*-values. **A** anterior default mode network, **B** posterior default mode network, **C** left central executive network, **D** right central executive network, **E** salience network, **F** auditory network.

**Fig. 5:**
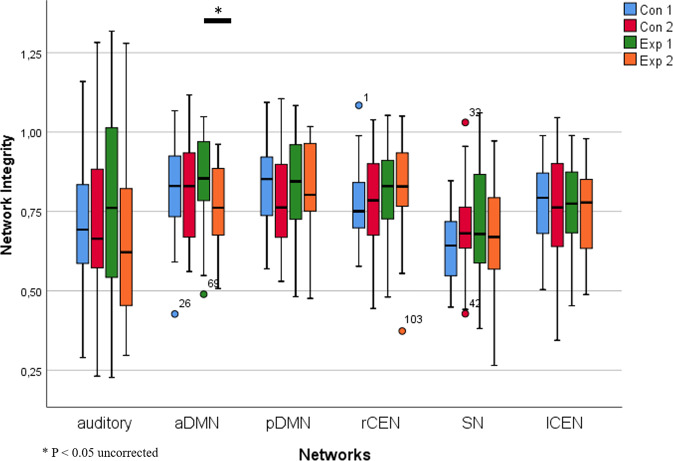
Integrity of fMRI-based networks. Distribution of integrity indices is shown as boxplots. Con 1: active control group at T1 (before training), Con 2: active control group at T2 (after training), Exp 1: experimental group at T1 (before training), Exp 2: experimental group at T2 (after training).

Figure 4 shows the RSNs of interest as extracted from the fMRI and PET data. ANOVAs revealed no significant *group* × *time* interaction for any network neither in the fMRI nor in the PET data. At an uncorrected threshold of *p* < 0.05, there was a change for the SN and aDMN in the fMRI data (Table 4 and Fig. 5), but not in the PET data (Table 5 and Supplemental Fig. S2).

**Table 4:** ANOVA for integrity of fMRI-based networks.

Network	EXP		CON		Interaction group × time	
	T1 M (SD)	T2 M (SD)	T1 M (SD)	T2 M (SD)	*F*_(1,53)_	*p*
Auditory	0.76 (0.29)	0.67 (0.25)	0.71 (0.19)	0.71 (0.23)	1.47	0.23
aDMN	0.85 (0.14)	0.77 (0.11)	0.81 (0.15)	0.82 (0.16)	5.05	0.03
pDMN	0.82 (0.17)	0.81 (0.16)	0.83 (0.14)	0.79 (0.15)	0.51	0.48
rCEN	0.81 (0.15)	0.82 (0.15)	0.78 (0.12)	0.77 (0.14)	0.13	0.72
SN	0.71 (0.18)	0.67 (0.17)	0.64 (0.11)	0.70 (0.13)	5.14	0.03
lCEN	0.77 (0.14)	0.75 (0.13)	0.78 (0.12)	0.75 (0.20)	0.10	0.75

*M* *(SD)* mean (standard deviation), *aDMN* anterior default mode network, *pDMN* posterior default mode network: *lCEN* left central executive network, *rCEN* right central executive network, *SN* salience network, uncorrected *p*-values.

**Table 5:** ANOVA for intergrity of FDG-PET-based networks.

Network	EXP		CON		Interaction group × time	
	T1 M (SD)	T2 M (SD)	T1 M (SD)	T2 M (SD)	*F*_(1,53)_	*p*
Auditory	−0.04 (0.90)	0.00 (1.11)	−0.06 (0.91)	0.10 (1.04)	0.57	0.45
aDMN	−0.09 (0.94)	−0.04 (1.13)	0.01 (0.82)	0.13 (1.05)	0.10	0.75
pDMN	−0.22 (0.80)	−0.17 (0.98)	0.19 (1.07)	0.21 (1.03)	0.03	0.85
rCEN	−0.26 (0.89)	−0.11 (0.93)	0.13 (0.97)	0.25 (1.10)	0.01	0.91
SN	−0.13 (0.92)	−0.13 (1.08)	−0.02 (0.85)	0.29 (1.06)	3.87	0.06
lCEN	−0.12 (0.94)	−0.07 (1.02)	0.09 (0.97)	0.11 (1.03)	0.04	0.84

*M (SD)* mean (standard deviation), *aDMN* anterior default mode network, *pDMN* posterior default mode network: *lCEN* left central executive network, *rCEN* right central executive network, *SN* salience network, uncorrected *p*-values.

### Tract-based spatial statistics

The two-way mixed-effect ANOVA for repeated measures showed no significant *group × time* interaction in any DTI map. Analyses with uncorrected thresholds *p* < 0.001 and *k* > 50 voxels did not reveal any significant clusters in any DTI map.

## Discussion

Following 8-week *n*-back training, neither near nor far transfer effects were detected in middle-aged adults, despite the presence of significant practice effects. Consistently, there were no significant changes in comprehensive analyses of multimodal neuroimaging data.

Given the inconsistent literature about transfer effects, we designed our study to address common limitations of the previous studies. To the best of our knowledge, the present work provides the most comprehensive assessment of neural correlates of WM training and transfer effects reported so far. Specifically, instead of relying on a single surrogate index of neural plasticity, we assessed five neuroimaging-based markers, at a voxel and network level. Along with testing cognitive changes with a broad test battery, this comprehensive and consistent pattern of results allows for stronger inferences than the previous studies. Second, as the mechanisms of transfer are still unclear, we isolated WM transfer effects from non-specific intervention effects by including an active control group. Specifically, because the control training was a non-adaptive version of the experimental WM training paradigm, we were able to distinguish transfer effects driven by training-induced WM gains from performance gains due to the acquisition of task-specific or stimuli-specific expertize, including material-specific strategies and learning. Third, we tested and closely supervised a larger-than average sample [69] of a typically neglected age group using an intensive, adaptive home-based intervention. Fourth, we evaluated transfer effects in cognition by combining two statistical approaches, frequentist and Bayesian statistics, thereby providing not only dichotomous information about the significance of effects, but also a continuous measure for the strength of evidence. Fifth, to reduce the likelihood of false positives, we rigorously corrected for multiple testing. Finally, with explorative purposes, we also analyzed the neuroimaging data using liberal significance thresholds without correction for multiple tests.

### Lack of evidence for transfer effects

In line with the results of the numerous studies, we found significant practice effects in the experimental group. Significant transfer effects were present only in one subtest of the nearest transfer category, the Digit Span forward. These effects were accompanied by strong Bayesian evidence. There was no corresponding improvement even in the Digit Span backward, a closely related subtest with the same stimuli. Neither near nor far transfer effects were detected. Of note, by transfer effects, we explicitly refer to improvements in an experimental group relative to an active control group. This definition allows the isolation of any cognitive gains *specifically due to* adaptive *n*-back training. Thus, WM training-related gains appear to generalize to performance in other WM tasks only to a very limited degree. Moreover, the gains are generalizable neither within the same domain nor to other cognitive domains.

### Lack of evidence for neuronal plasticity in the neuroimaging data

To the best of our knowledge, this is the first study of WM using multi-modal neuroimaging including PET. Surprisingly, there are few investigations of the neural substrates of WM training. Different from the present study, in which we did not detect any changes in ALFF or glucose metabolism after WM training, Takeuchi et al. reported increased brain activity in the dorsal prefrontal cortex following WM training [27]. However, the authors did not include an active control group and, therefore, these changes could reflect test-retest or non-specific intervention effects. So far, only two studies have explored an impact of WM training on RSN connectivity, with findings of both increased and decreased connectivity within the DMN, as well as between the DMN and central executive network (CEN) [28, 29]. Here, we did not find any robust training-induced changes in the DMN, CEN or other established neurocognitive RSNs. Only exploratory post hoc tests revealed reduced integrity of the aDMN in the experimental group. However, this observation did not survive multiple correction or contrasting against the active control group. Notably, neither of the past studies included an active control group. While there has been no TBSS study on the effects of WM training, a few research groups have explored effects of this kind of training on white matter integrity using voxel-based morphometry or an ROI analysis [30–33]. They reported training associated-changes in frontal and parietal white matter tracts [33], corpus callosum [31], in regions of the dopaminergic system [32], and in the superior and inferior longitudinal fasciculus [30]. However, in line with our resting-state fMRI and FDG-PET results, we found no significant effects of WM training on white matter integrity. Again, the above studies included either no control group [33] or a passive control group only [31, 32].

### The need for an active control group

As discussed elsewhere (e.g., [7]), there are large methodological differences between WM training and transfer studies, for example, variations in training tasks (e.g., *n*-back vs. complex span tasks), training conditions (duration and frequency), and age of participants. However, the key factor contributing to the inconsistent findings in previous behavioural and neuroimaging studies is the presence and type of a control group (passive or active). Critically, meta-analyses that explicitly distinguish between the types of a control group typically report a lack of far, sometimes also near, transfer effects [5, 15, 17].

Alternative interventions administered to active control groups can either involve entirely different activities, such as completing questionnaires rather than cognitive tasks (e.g., [70]), adaptive cognitive tasks that differ from the experimental group but follow the same adaptive procedures, such as adaptive visual search training (e.g., [20]), or, as in the present study, non-adaptive versions of the experimental training intervention. Adaptive alternative interventions arguably control better for motivational appeal and believability than non-adaptive alternative interventions (see also ref. [18]); however, administering non-adaptive active control training allows for keeping the tasks and materials consistent across training groups. Specifically, the higher the similarity between active control and experimental group training, the more stringent the control of transfer gains driven by task- or material-specific expertize when contrasting the groups over time (see also ref. [71]). In the present study, we chose a non-adaptive control group to allow for isolating transfer effects due to training-induced increases in WM capacity from improvements in WM efficiency due to the acquisition of task- or material-specific expertise.

In a recent meta-analysis by Sala at el. [4] 8 out of 43 reviewed studies assessed transfer and included a control group performing the same tasks as the experimental group. Critically, no transfer effects were observed in 3 out of 8 studies [72–74], and only nearest transfer effects as measured with digit span forward and/or backward were observed by three other studies [19, 75, 76]. Near and far transfer effects were reported only by Brehmer et al. [77] for the PASAT task requiring sustained attention and by Simon et al. [78] for the Digit Symbol task requiring psychomotor ability, sustained attention, processing speed and, to a lesser degree, WM. Of note, these are two transfer tasks that we did not include. Similar findings were reported by Aksayli et al. [79], who evaluated the effects of adaptive Cogmed WM training, a commercial training programme, relative to non-adaptive Cogmed training. The authors reported a consistent lack of far transfer, small to medium effects sizes for near transfer and that these transfer effects depended on the similarity of the transfer tasks to the WM training paradigm. This is in line with our results, supporting the view that WM training-related gains appear to generalize to performance in other WM tasks only to a very limited degree. Indeed, when exploring within-group changes from pre-test to post-test, we observed significant gains in all three transfer categories, including far transfer, although these did not survive correction for multiple tests. Importantly, however, the experimental group did not improve over and above the changes observed in the control group. Surprisingly, we also observed a decline in the Digit Span Forward (nearest transfer) in the control group, which may have contributed to the only significant transfer effect in the main analysis. This implies that both training interventions, no matter how specific they have been designed, can trigger performance improvements which are not due to an increased WM capacity, but most likely due to non-specific cognitive training effects, such as improved attentional processing [19], or due to the acquisition of strategies such as chunking or visualization [80]. These strategies are likely paradigm-specific and, thus, could be applicable to both the experimental and active control group, thereby also explaining the cognitive improvements in the control group. In this context, we propose the term *pseudo-transfer effects* to describe such gains that are unrelated to increases in WM capacity and also not accompanied by strong neuroplastic effects as measured by, for instance, DTI. This explanation is in line with the only nearest transfer effect that we observed in the experimental group contrasted against the active control group. As there was no transfer to any other task, it is highly unlikely that the effect was driven by increased WM capacity. Instead, we argue that the observed improvement in the forward Digit Span, a measure of verbal short-term memory, more likely is a pseudo-transfer effect imparted by co-engagement of short-term memory and acquisition of effective strategies during adaptive *n*-back training. As transfer effects within and between cognitive domains were shown to be of equivalent magnitude when comparing different WM training paradigms (e.g., n-back training and complex span training) [81], it is reasonable to assume that our results are generalizable to other WM training paradigms.

Overall, the present results, in particular on the neuroimaging side, may serve as a useful reference for future cognitive training studies not only in healthy individuals, but also in patients with neuropsychiatric disorders. While patients with affected WM and neuroimaging indices of brain health at baseline might potentially have more space for gains then healthy subjects, commonly co-existing attention deficits and disease-related deterioration of neural plasticity may limit efficiency of WM training in patients. We suggest that future training studies should target multiple cognitive domains beyond WM and include neuroimaging techniques beyond fMRI, DTI, and FDG-PET.

## Conclusion

In this prospective, randomized, actively controlled, and single-blind study, we provide strong and consistent evidence for the absence of near and far transfer effects following 8-weeks of adaptive WM training in healthy middle-aged adults, despite a pronounced improvement in the *n*-bask training task. Repeated multimodal imaging revealed no training-induced changes in resting state fMRI, DTI, and FDG-PET data. More specifically, comprehensive analyses of putative markers of neuronal plasticity in terms of white matter integrity, BOLD signal, and glucose metabolism, at a voxel and network level, failed to discover significant alterations. We propose the term “pseudo-transfer effects” to characterize gains in a trained task, resulting from co-engagement of non-targeted cognitive domains and/or improved efficiency in using the targeted cognitive capacity due to acquisition of new strategies. Critically, we argue that such pseudo-transfer effects do not reflect increases in WM capacity. Instead, the presently observed practice effects may be a result of optimized task processing strategies, which do not necessarily engage neural plasticity.

## Supplementary information

Supplemental material

## Data Availability

The data that support the findings of this study are available from the corresponding author on reasonable request. In this study, all participants have signed an informed consent form approved by the local ethic committee stating that their data will only be made accessible to a third person for the purpose of clinical examination.

## Code availability

No new code was developed for generating the results. All results were obtained by functions belonging to publicly available software. The used software and/or functions are stated at an appropriate location in the manuscript in the “Methods” section.
