# Supplementary material for: Adaptive working memory training does not produce transfer effects in cognition and neuroimaging
Source: Transl Psychiatry. 2022 Dec 13;12:512. doi: 10.1038/s41398-022-02272-7 (PMC9747798; doi:10.1038/s41398-022-02272-7)
Supplement: Supplementary file 1 — Supplemental material [file 41398_2022_2272_MOESM1_ESM.docx]

# Imaging data acquisition

## Positron emission tomography

PET data were acquired for 60 min in list mode with a craniocaudal scan direction and a z-axis distance of 26 cm. PET image reconstruction was performed offline using Siemens e7 Tools (Siemens Molecular Imaging, Knoxville, USA). Ordered subsets expectation maximisation (OSEM) algorithm with 3 iterations of 24 subsets with a 5 mm Gaussian filter, to eliminate the high frequency noise, was used. The resulting PET images had a matrix dimensions of 344 × 344 with 127 sagittal slices with a reconstructed voxel size of 1.04 × 1.04 × 2.03 mm^3^. Tissue segmentation based magnetic resonance attenuation correction (MRAC) was performed using an ultra-short time of echo (UTE) μ-map which was acquired with the following parameters: 300 sagittal slices; voxel size = 1.6 × 1.6 × 1.6 mm^3^; matrix size = 300 × 300 mm.

## Structural T1-weighted MRI

Structural T1-weighted (MPRAGE) images were acquired using a three-dimensional (3D) normal gradient recalled sequence with the following parameters: 160 sagittal slices; time of repetition (TR) = 2300.0 ms; echo time (TE) = 2.98 ms; flip angle = 9.0º; field of view (FOV) = 256mm; bandwith = 240 Hz/Pz; matrix size = 256 × 240 mm; slice thickness = 1.0 mm (no gap) and voxel size = 1.0 × 1.0 × 1.0 mm^3^ voxel size.

## Resting state fMRI

FMRI data were acquired using an echo planar imaging pulse (EPI) sequence with the following parameters: 40 slices; 212 volumes; TR = 2230 ms; TE = 30 ms; flip angle = 90 º; FOV= 192 mm; bandwith= 2232 Hz/Px; matrix size 192 x 144 mm; slice thickness = 3.0 mm (0.6 mm gap); 3.0 x 3.0 x 3.0 mm^3^ voxel size and interleaved acquisition.

## Diffusion tensor imaging

DTI data were acquired using an EPI sequence with the following parameters: 60 slices; 30 volumes; TR = 10800 ms; TE = 82 ms; flip angle = FOV= 260mm; bandwidth= 1672 Hz/Px; matrix size = 260 ×120; slice thickness = 2 mm (no gap) and 2.0 × 2.0 × 2.0 mm^3^ voxel size. Diffusion weighting was performed in multi-directional diffusion weighting (MDDW) mode along 30 diffusion gradient directions with b= 800 s/mm^2^ complemented by one image without diffusion weighting (b= 0 s/mm^2^). We used bipolar diffusion gradients to reduce motion artifacts.

## Spatially constrained ICA

A spatially constrained ICA is a semi-blind ICA algorithm which uses prior information about sources as reference signals (i.e. computed spatial maps from the fMRI data), enabling to extract only the desired sources ^1^. The resulting independent components have higher SNR ratio than traditional ICA algorithms ^1^. Applying a spatially constrained ICA to FDG-PET data ensured a high cross modality network similarity leading to an accurate comparison of training effects on equivalent but modality specific derived networks. We chose to apply components extracted from fMRI data as reference spatial maps because well-established RSN templates from fMRI data exist, which were used for RSN identification ^2,3^.

# Voxel-wise analyses of the ALFF and PET data

Figure S 1: Explorative ANOVA of group by time interaction of voxel wise ALFF (A) and FDG-uptake (B) at p < 0.001 with a cluster extent of k > 50 voxels.

A


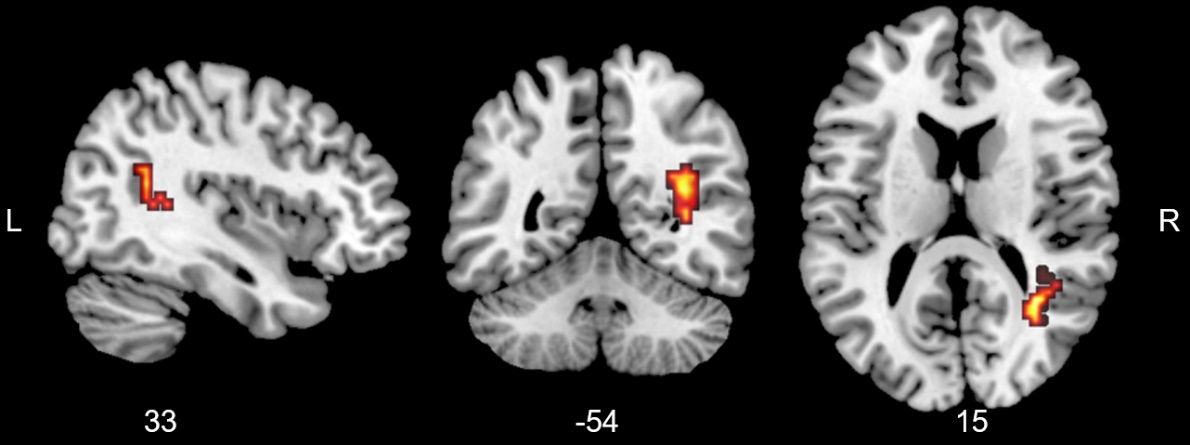

*Spontaneous activity decreased in experimental group than control group in right angular gyrus (extending to superior temporal gyrus) over time. Cluster size: 74 voxels; L, left; R, right.*

B


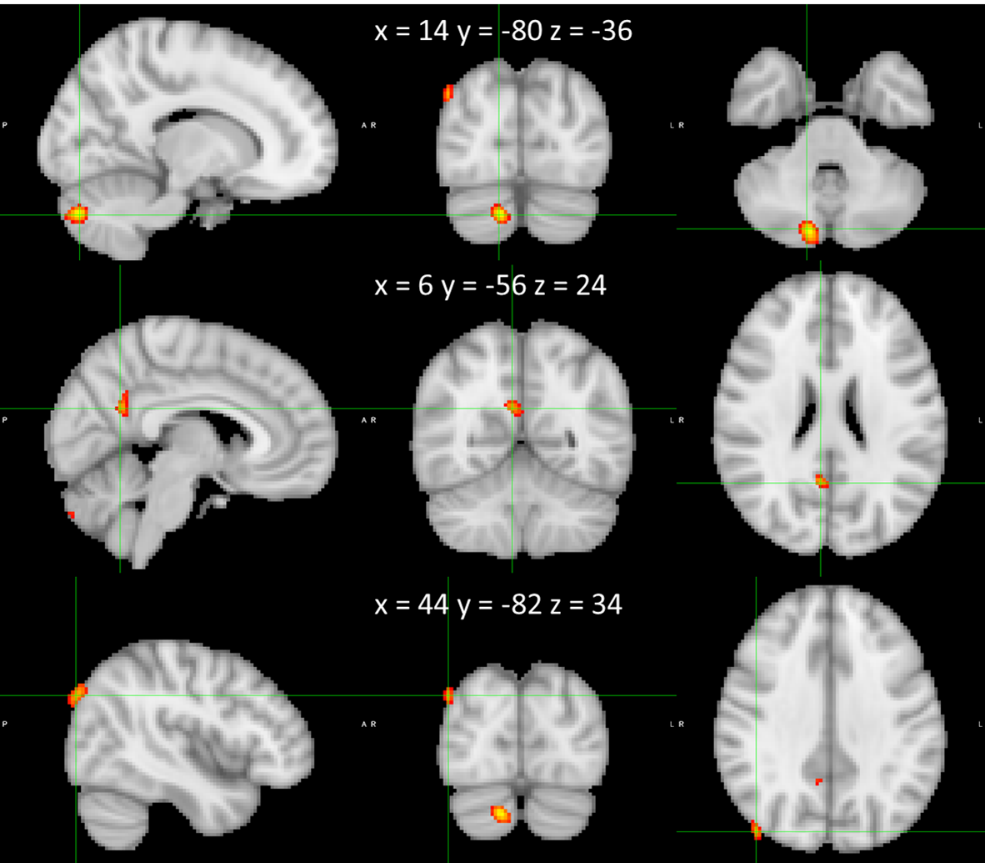


*Higher rFDG-uptake in experimental group compared to the control group over time (Exp (T1 < T2) > Con (T1 < T2)).*

At an uncorrected level (p < 0.001, k > 50 voxels), one cluster located in the right angular gyrus showed decreased ALFF values in the experimental group compared to the control group after training (Figure S1 A). Of note, around 45% (34/74) of voxels in this cluster are white matter. For FDG-PET, at an uncorrected level (p < 0.001; k > 50) two cluster showed higher FDG-uptake in the experimental group compared to the control group over time (Figure S1 B). One additional cluster was located outside of the brain and was therefore considered noise.

# Network analyses of the resting state PET data

Figure S 2: Integrity of PET-based networks. Distribution of integrity indices is shown as boxplots. Con 1: active control group at T1 (before training), Con 2: active control group at T2 (after training), Exp 1: experimental group at T1 (before training), Exp 2: experimental group at T2 (after training).


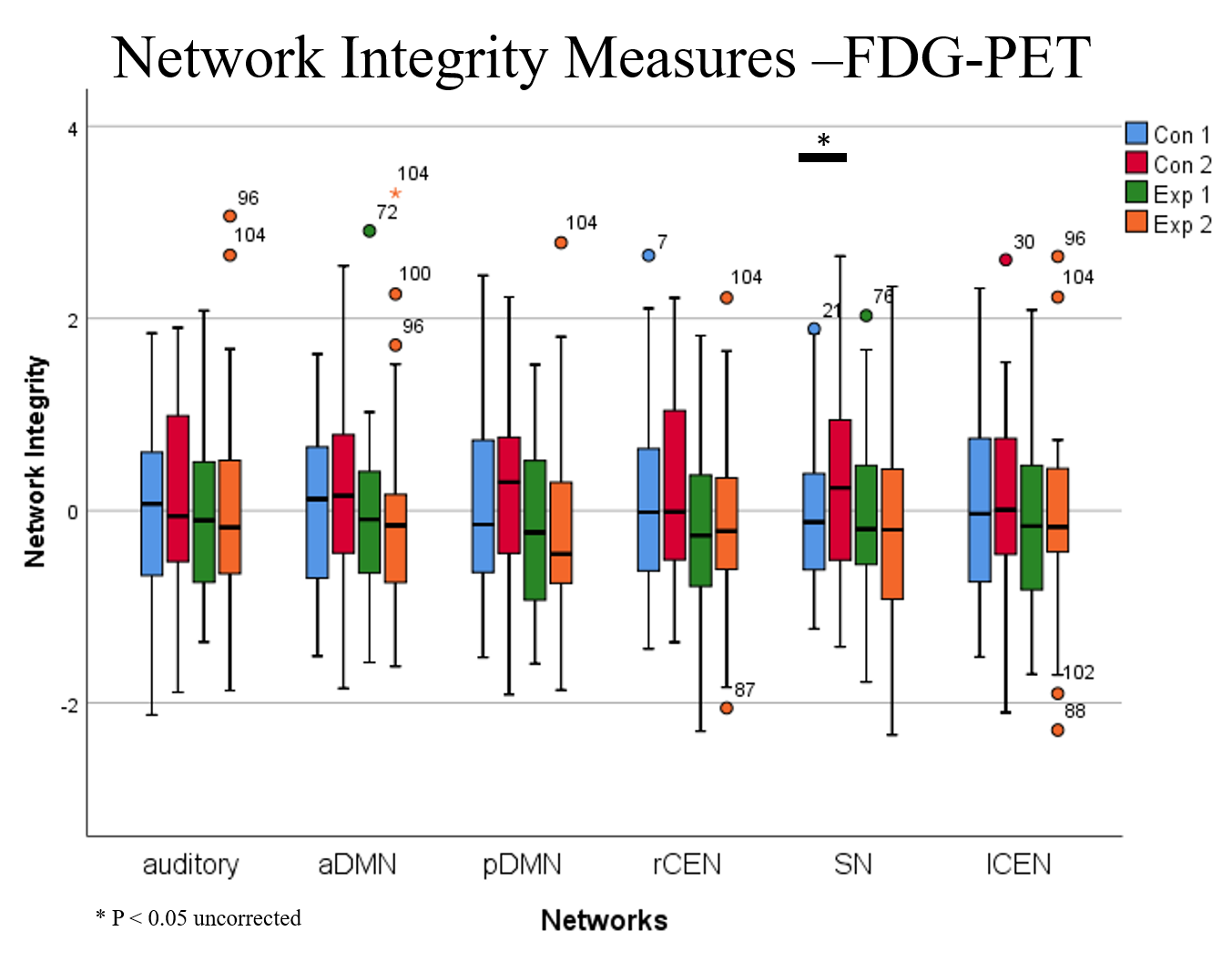


# References

1. Lin, Q.-H., Liu, J., Zheng, Y.-R., Liang, H. & Calhoun, V. D. Semiblind spatial ICA of fMRI using spatial constraints. *Hum. Brain Mapp.* **31**, 1076–1088 (2010).

2. Allen, E. A. *et al.* A baseline for the multivariate comparison of resting-state networks. *Front. Syst. Neurosci.* **5**, 2 (2011).

3. Yeo, B. T. T. *et al.* The organization of the human cerebral cortex estimated by intrinsic functional connectivity. *J. Neurophysiol.* **106**, 1125–1165 (2011).
